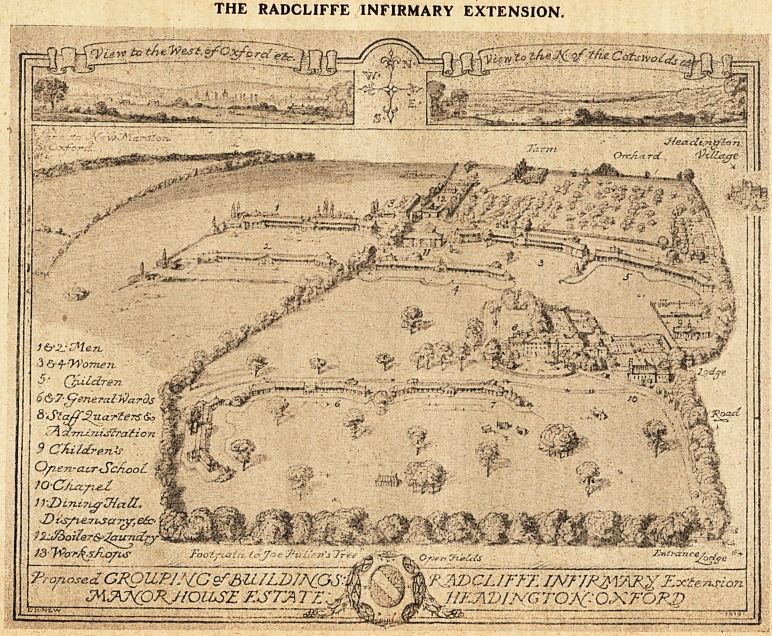# Oxford's Great Memorial to Valiant Men

**Published:** 1919-11-15

**Authors:** 


### RADCLIFFE INFIRMARY AND COUNTY HOSPITAL.

### OXFORD'S GREAT MEMORIAL TO VALIANT MEN.

THE RADCLIFFE INFIRMARY EXTENSION.

FOR many years the curative side of the Radcliffe Infirmary and County Hospital, Oxford, has been carefully tended and brought up to the standard of modern scientific requirements, but little provision has hitherto been made either for the preventive side of Medicine or for a Maternity Department. From an estimate made of the total number of beds required for peace-time work, there is a general shortage throughout the country, and this hospital will require at least another 150. The beds in the

**Radcliffe Infirmary and County Hospital**—(*continued*).

women's wards have not been sufficient for several years to meet the demands made upon them, and the waiting-lists are so long that many months elapse before the less urgent cases can be admitted.

The children's wards are unsuitably placed for their work and better and more speedy results would be assured if they were wholly removed, except for a small reception ward, from their present site on to higher and more open ground.

SITE FOR EXTENSION.

To meet these needs, extension on the present site is neither possible nor practicable, and the opportunity occurring for the purchase of a house with grounds of 140 acres, within two miles of the centre of Oxford, suitable in every respect for the purpose, the Committee of Management decided to acquire the Manor House Estate, Headington, for the purpose of extending the hospital; and to lease a house in Parks Road for a Maternity Department. Both these proposals have received the approval of the Court of Governors. The new site will not only render possible the extension that is immediately necessary, but will also make provision for many years to come.

IMPROVING THE HOSPITAL FACILITIES FOR OXFORD AND DISTRICT.

An important scheme signed by the Rev. George B. Cronshaw, Treasurer of the Hospital, has been formulated for enlarging and improving the hospital and convalescent facilities for Oxford and district. This scheme is designed as a memorial to the gallant men who gave their lives during the war, and it is felt that no nobler or more lasting memorial could be raised than one which ensures greater health and vigour to those who remain.

OXFORD'S SUCCESS AS A LEADING MEDICAL CENTRE.

During the war more than 40,000 wounded and sick soldiers were treated in Oxford; and the success of Oxford as a leading medical centre during this period was in no small measure due to the foresight of the Committee of Management of the Radcliffe Infirmary in making more adequate provision for the teaching of operative surgery, in establishing a laboratory for pathology, and in founding and equipping departments for dentistry and for electro-therapeutics and *x*-ray. More recently two new special departments have been founded—one for orthopædic surgery, to deal with the deformities due to war injuries and to provide special skilled help to civil workers and crippled children; and the other for the special treatment of cases of neurasthenia, to alleviate and cure this distressing condition and prevent its unfortunate victims from drifting into the stage of insanity. A large number of war pensioners are also suffering from tuberculosis, and open-air treatment is an urgent necessity for them.

NEW MATERNITY DEPARTMENT.

A new Maternity Department has been organised, and will be opened in a few weeks, to advise expectant mothers and to deal with the more difficult cases of childbirth, to form a centre for the education of nurses and midwives, and to perfect the infant welfare department which has been in existence for several years.

OPEN-AIR WARDS FOR CONSUMPTIVES.

Open-air wards will be erected without delay for the resident-care of cases of consumption, and ample accommodation provided for the needs in this respect of the County and City of Oxford, and possibly for the Borough of Reading. At present, other than the Radcliffe Infirmary, there is no such institution under the direct charge of the Insurance Committees of Oxford and Oxfordshire. Not only have the early cases of consumption to be provided for, but also the surgical cases, and the children can be more advantageously treated on a site affording abundance of open air and sunshine. Accommodation for the advanced cases is also sadly needed, both for the comfort of the patients themselves and also to obviate what now so frequently occurs, *i.e.* an advanced case living in the midst of a young and growing family in a small cottage and so spreading the disease.

NEW SITE AT HEADINGTON.

The new site at Headington is an ideal one for the hospital extension. It is situated on high ground, the subsoil is exceptionally dry, and it is freely open to the sun and air. In addition, it is conveniently situated near the railway centre for the district, an advantage which will make for economy in administration and for accessibility to the patients and their friends.

SAFEGUARDING THE NATIONAL HEALTH.

Never has the importance of safeguarding the national health been so great as it is to-day, when the population is suffering from the strain of five years' unexampled warfare with the loss of so many of its finest men. It cannot be doubted that the proposed additions to the hospital will prove of very great value in this respect: in raising the standard of health in our district and in contributing towards the greatest asset any nation can possess—a fit and vigorous people. Mr. G. Randall Higgins and Mr. T. H. Rose have generously started the appeal Fund with a donation of £1,000 each; the Medical Staff have given 120 guineas. A further donation of £15,000 has been promised by the British Red Cross Society specially for the new tuberculosis department, with the condition that a similar sum be raised locally. It is earnestly hoped that £50,000 will be raised, so that the hospital may be able to claim this donation without delay.

UNITED TO WIN SUCCESS.

Oxford has in the past loyally supported the Radcliffe Infirmary in its efforts to meet the new calls that must inevitably be made on any hospital determined to keep pace with modern requirements; and it is with confidence begotten of past support that this new appeal is put forward on its behalf.

**Subscriptions and donations to the above appeal may be sent to the Treasurer, The Radcliffe Infirmary and County Hospital, Oxford, or paid into Messrs. Barclays, Ltd., Old Bank, Oxford.**

## Figures and Tables

**Figure f1:**